# Synthesis of titanium dioxide nanoparticles with renewable resources and their applications: review

**DOI:** 10.55730/1300-0527.3443

**Published:** 2022-05-12

**Authors:** Umut Şafak ÖZTÜRK, Alime ÇITAK

**Affiliations:** Department of Chemical Engineering, Faculty of Engineering and Architecture, Eskişehir Osmangazi University, Eskişehir, Turkey

**Keywords:** Titanium dioxide, nanoparticles, green synthesis, dye removal, photocatalysis

## Abstract

Metal-oxide nanoparticles have reached a wide range of applications in the last ten years. Titanium dioxide nanoparticles stand out with their unique crystal structure and near-perfect physical and chemical properties at nanoscales (crystal size between 10–100 nm). It has created many applications with its white pigment, semiconductor state, and effective photocatalytic properties, but the synthesis of these nanoparticles is very damaging to nature. Titanium dioxide nanoparticles, which can be synthesized with toxic solvents or high-energy machines, have started to be synthesized by the green synthesis method, which has been a cheap and easy method in recent years. The application areas of these nanoparticles synthesized with renewable resources (leaves, roots, seeds, etc.) are increasing day by day. This article gives information about the synthesis of titanium dioxide nanoparticles with renewable resources and the application of these nanoparticles synthesized by means of chemical and green synthesis methods.

## 1. Introduction

Nanomaterials are materials with at least one of their dimensions less than 100 nm (1 nm = 10^−9^ m). As the size of the compounds decreases, they begin to show near-perfect properties. For example, as the size of the graphene nanomaterial decreases, it becomes highly conductive [1]. There are different dimensions of nanomaterials, nanocrystals (quantum dots), nanotubes, nanosheets, and nanowires. Nanotechnology application areas have several usages: cosmetics [2], solar cells [3], protective coating [4], pharmaceutical industry [5], cancer and tumour cell detection [6], dye pigments [7], wastewater treatment with carbon nanoparticles [8], making electrochemical biosensors with gold nanoparticles [9], the use of iron-based nanomaterials as anodes in the lithium battery [10] and the use of nanoparticles in the anticancer activity tests of ZnO [11].

Like metal oxides, titanium dioxide (TiO_2_) has a wide range of applications; it is a compound commonly used in products such as dye colours, coatings, solar energy, and toothpaste. Titanium dioxide singularly shows a unique crystal structure as rutile, anatase, and brookite. Industrial production was first started in Norway, America, and Germany in 1918 [12]. Titanium dioxide nanoparticles are used effectively due to their very high photocatalytic properties. It causes degradation in organic pollutants by radicalizing hydroxyl groups in water molecules exposed to UV light. Also, TiO_2_ is an effective semiconductor, based on this, many new studies are being recorded in the field of photocatalytic dye reduction [13]. Prorokova et al. [14] tried the antibacterial properties of silver-doped titanium dioxide nanoparticles on polyester fabric, and they became successful. Deshmukh and his students [15] successfully removed methylene blue with the titanium dioxide nanoparticles they synthesized. Kuriechen and Murugesan [16] achieved nearly successful results of 80% in the study of using carbon-doped titanium dioxide nanoparticles in reducing azo dyes.

Today, two primary methods are used in nanomaterial synthesis as physical and chemical. However, these two methods bring many problems. Firstly, large areas and high energy consumption are required for physical methods (crushing, lithography, etc.). The biggest problem with the second method for the chemical method is using toxic solvents and toxic chemicals. The third method, the green synthesis method, is quite different from the other two methods. Green synthesis is simple, inexpensive, and eco-friendly compared to the methods described above. In general, bacteria, fungi, or plants are used in green synthesis methods. High efficiency and low toxicity have been achieved by replacing some basic materials (solvent or initiator) in the chemical synthesis method with biological material. In recent years, scientists have turned to this field and have achieved successful applications [17]. Raliya and Tarafda [18] succeeded in synthesizing titanium dioxide, zinc oxide, and magnesium oxide nanoparticles from Fusarium oxysporum fungus. Marimuthu and his students [19] obtained titanium dioxide nanoparticles, 10 nm in length, which they synthesized with Calotropis gigantea flower. There have been many nanoparticles syntheses like this.

### 1.1. Physical and chemical properties of titanium dioxide

**Figure 1:**
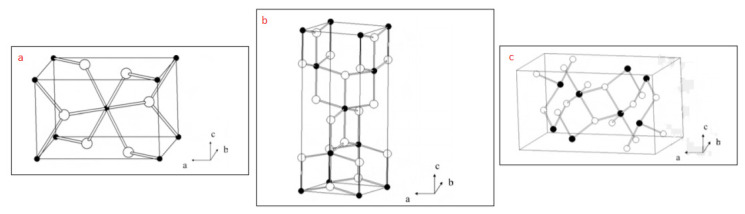
The appearance of the crystal lattice structures of titanium dioxide. a) Rutile form b) Anatase form c) Brookit form [20]

Titanium dioxide is also known as titania. The formula of titania with a molecular weight of 79.89 g/mol is TiO_2_ (O = Ti = O). Its physical appearance is in the form of a white powder, and its melting point is approximately 1750 °C. Titanium dioxide has three different crystal structures. Therefore, it can show other physical properties. These are called rutile, anatase, and brookite (Figure 1). Rutile is the most thermodynamically stable crystal form of these [20].

For this reason, brookite and anatase turn into rutile forms at very high temperatures [20]. Conversion from anatase to rutile is exothermic and generates 12.6 kJ of heat per mole. Compared to rutile, anatase has a lower Mohs hardness [21]. Therefore, anatase is predominantly used in cases where its lower hardness has a technical advantage. Titanium tends to form highly stable oxides [21]. Consequently, titanium dioxide is hardly reactive. Titanium dioxide is insoluble in water. Calcined titanium dioxide does not dissolve even in hot and concentrated acids. However, it can be dissolved in molten acids and bases. Therefore, it is used in beam-refracting glasses [20].

Titanium dioxide, like other nanomaterials, begins to show near-perfect properties when at least one dimension is between 1–100 nm. Titanium dioxide can be synthesized as nanosheets, nanofibers, nanotubes, nanoparticles, and nanocomposites. High photocatalytic properties increase the usage range of titanium dioxide nanoparticles (TiO_2_-NP) [21].

### 1.2. Photocatalytic properties of titanium dioxide

Titanium dioxide is a potent photocatalyst. The working principle of photocatalysts is based on the excitation of semiconductor material with light energy equal to or greater than the material’s bandgap energy. An electron in an electron-filled valence band (VB) is excited upon irradiation to an empty conduction band (CB), leaving a positive hole (h +) in VB. These electrons and holes (e− and h +) are mainly responsible for forming active species that degrade target molecules, and they cause reduction and oxidation, respectively [22]. As a semiconductor mechanism, TiO_2_ exposed to UV-ray (<390 nm) in Equation 1 forms an electron (e-) and a positive hole (h+). In Equations 2 and 3, the solid positive spot separates the water molecule to form a radical hydroxyl (OH •) group, and in Equation 4, the electrons make the oxygen radical. This radical hydroxyl group begins to circulate in water. Finally, in Equation 5, the radical hydroxyl group is oxidized with organic pollutants (dyes, pesticides, etc.), and carbon dioxide and water are formed. Thus, the degradation of these pollutants occurs. The photocatalytic activity of semiconductor metal-oxides is based on these equations (below) [22].


(1) $${\text{TiO}}_{2}+\text{h}\nu \;(\lambda <390\;\text{nm})\to {\text{TiO}}_{2}(\text{eCB}-+\text{hVB}+)$$

(2) $$\text{h}++\;{\text{H}}_{2}\text{O}\to \text{H}++\;\text{OH}-$$

(3) h++ OH-→ OH•

(4) $$\text{e}-+\;{\text{O}}_{2}\to \;{\text{O}}_{2}•$$

(5) $$\text{Organic waste}+\text{OH}•+\;{\text{O}}_{2}\to {\text{CO}}_{2}+{\text{H}}_{2}\text{O}+\text{waste product}$$

### 1.3. Antioxidant effect of titanium dioxide

Titanium dioxide nanoparticles also show antioxidant properties [23]. This feature becomes effective as the grain size decreases like other photocatalytic properties. Three different measurements are performed: Dimethyl sulfoxide (DMSO) test, total phenolic test, and DPPH (2,2-diphenyl-1-picrylhydrazyl) methods. In general, titanium dioxide shows a high antioxidant effect on ascorbic acid, while the DPPH test measures how much titanium dioxide nanoparticles can scavenge free radicals [23].

### 1.4. Characterization of titanium dioxide nanoparticles

There are specific basic measurements in the characterization of nanoparticles. Analyses such as XRD, FE-SEM/EDS, FT-IR, UV-vis, and Brunauer-Emmett-Teller (BET) method provide basic information about the synthesized nanoparticles. XRD analysis can determine if titanium dioxide is anatase, rutile, or brookite and, helps in calculating particle size. Analysis of surface functional groups of titanium dioxide nanoparticles synthesized with green synthesis is an important step; for this purpose, FT-IR is used [24]. If titanium dioxide can be used as a photocatalyst, it must absorb light at a specific wavelength. To select this light source, it is necessary to measure at which wavelength titanium dioxide reacts in the UV spectrum [25]. Surface area and pore volume are determined by nitrogen adsorption/desorption using the Brunauer-Emmett-Teller BET method [26]. Analyses of the morphology, aggregation shapes, and sizes of nanoparticles are made using FE-SEM/EDS [27].

## 2. Chemical synthesis methods of titanium dioxide nanoparticles

Many different methods are used to synthesize titanium dioxide nanoparticles, such as the sol-gel method, hydrothermal method, solvothermal method, direct oxidation method, chemical vapor deposition, physical vapor deposition, electrochemical anodization, and biological synthesis. The most used of these rich synthesis methods is the sol-gel method [28].

### 2.1. Sol-gel method

**Figure 2:**
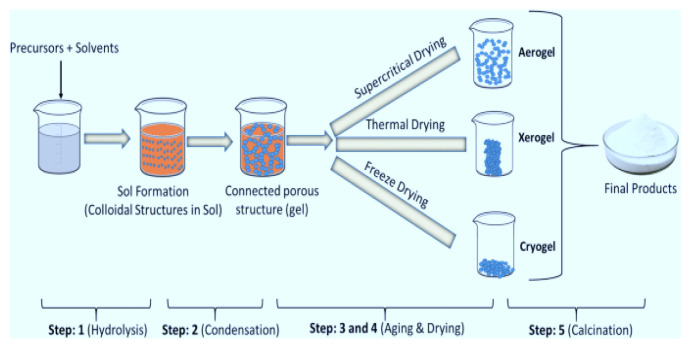
Visual representation of the sol-gel method.

The sol-gel method is frequently used to synthesize tin oxide, tungsten oxide, zinc oxide, and titanium dioxide nanoparticles. This method consists of five main lines; hydrolysis, polycondensation, aging, drying, and thermal decomposition. In the hydrolysis step, an initiator (metal alkoxide) and a solvent (alcohol, water, or solvent with hydroxyl bond) are required. At this stage, the aim is to separate the metal from the alkoxide and bring it together with the -OH bond. In polycondensation, the M-OH compound is freed from the solvent, and the M-O-M bond is formed. It is then left to age. Different gel forms are included according to different drying types, such as supercritical drying, thermal drying, freeze-drying, and finally, the product is obtained by calcination [22]. The sol-gel method for titanium dioxide nanoparticles is based on the hydrolysis reaction followed by condensation of a titanium (IV) alkoxide initiator. Hydrolysis with a small amount of water is preferred to obtain the Ti-O-Ti chain. Titanium tetraisopropoxide is generally preferred as the initiator. The rate constant for solidification and the viscosity of the solution depends on the temperature. The radius of the average TiO_2_ increases over time by the Lifshitz-Slyozov-Wagner model. The sol-gel method synthesizes titanium dioxide in the form of anatase [29]. This method is explained visually in Figure 2.

The hydrolysis of metal alkoxide in the sol-gel method applied for metal oxide nanoparticles is given in Equation 6. Then, the metal hydroxyls shown in Equation 7 begin to gel by forming the M-O-M bond. After the calcine, MOx/2 is obtained. The total equivalence of Equation 6 and Equation 7 is shown in Equation 8 [30].


(6) $$\text{M}(\text{OR})\text{x}+{\text{nH}}_{2}\text{O}\to \text{M}(\text{OR})\text{x-n}(\text{OH})\text{n}+\text{nROH}$$

(7) $$2\text{M}(\text{OR})\text{x-n}(\text{OH)n}\to (\text{OH})\text{n-}1(\text{OR})\text{x-n}-\text{M}-\text{O}-\text{M}-(\text{OR})\text{x-n}(\text{OH})\text{n-}1+{\text{H}}_{2}\text{O}$$

(8) M(OR)x+x/2H2O→MOx/2+xHOR

### 2.2. Hydrothermal method

Hydrolysis of metal salt solution can synthesize many nanoparticles, such as a wide variety of chemical compounds, including oxides, sulfates, carbonates, phosphates, and sulfides. Hydrothermal synthesis is usually carried out by reaction in aqueous solutions under controlled temperature and controlled pressure in steel pressure vessels called Teflon lined or autoclaves. According to the hydrothermal synthesis method, bypassing the gel step of the sol-gel process, after the hydrolysis reaction of metal-alkoxides in an alcohol-water environment, it is dried to room temperature and directly calcined to obtain solid MOx. Nanoparticles of desired particle sizes and morphologies are synthesized by controlling parameters such as temperature, pH, reactant concentrations, and additives [30].

### 2.3. Solvothermal method

The main difference between this method from the hydrothermal method is that the solvent is nonaqueous. Other than that, the steps are almost the same. The advantage of being anhydrous is that it can operate at high temperatures since high boiling point organic solvents can be selected. Compared to the hydrothermal method, the size, shape, and crystallinity of TiO_2_-NPs can be controlled better [31].

### 2.4. Direct oxidation method

Oxidants are used for the oxidation of titanium metal. The direct oxidation method is generally used for the synthesis of TiO_2_ nanorods. This method takes place by the dissolution precipitation mechanism. For example, when the anodized titanium plate is heat-treated at 500 °C for 6 h in an oxygen environment, crystallized TiO_2_ nanotubes are obtained. It has also been found that direct oxidation of titanium metal with hydrogen peroxide leads to the formation of TiO_2_ nanorods. Acetone and pure water can be used as oxygen sources for the oxidation of titanium metal. Acetone is a good source of oxygen, and when used at high temperatures, well-aligned, and highly dense nanorods can be synthesized [30].

### 2.5. Chemical and physical vapor deposition methods

Vapor deposition methods are a method obtained as a solid phase material by condensing a vaporized material. This method is generally used to make coatings change the mechanical, electrical, thermal, optical, corrosion resistance, and wear resistance of various substrates. However, it has recently been used frequently in the synthesis of various nanomaterials. Steam accumulation takes place in a vacuum environment. If a chemical reaction does not occur, it is called the physical vapor deposition method. With this method, TiO_2_ nanofilms at 30 nm and TiO_2_-NP at 10 nm were synthesized [30].

### 2.6. Electrochemical method

The primary purpose of this method is to create an oxide layer on the metal surface. It uses titanium as the anode part of an electrical circuit and forms the oxide layer on this anode. Anodization changes the microscopic texture of the character and the metal’s crystalline structure close to the surface.

Titanium is set as the anode to be oxidized, and a conductor such as a cathode and platinum is selected. When the reaction starts, it withdraws metal electrons positively, so the anode side reacts with water to form a dense oxide layer. Thus, a nanoporous titanium dioxide layer is formed.

### 2.7. Sonochemical method

Ultrasound is used to synthesize many nanostructured materials such as transition metals, alloys, colloids, and carbides. The chemical effects of ultrasound do not work molecularly. Instead, it makes use of cavitation. TiO_2_ nanomaterials were synthesized with this method.

Electromagnetic waves ranging from 0.3 to 300 GHz and with a wavelength of 1 mm to 1 m are used in microwave-assisted methods. Any material containing moving electric charges, such as conductive ions, is heated by microwaves. As polar molecules try to orient with the rapidly changing alternating electric field, heat is generated by the molecules’ rotation, friction, and collision. If ions are present in the solution, they move through the solution and constantly change direction depending on the direction of the electric field, resulting in local temperature rise due to friction and collision. Therefore, the microwave heating method provides a shorter reaction time, higher reaction rate, and higher efficiency than conventional heating methods [31].

## 3. The green synthesis method of titanium dioxide nanoparticles

Titanium dioxide nanoparticles are the last one of the popular topics with renewable resources in recent years. Various studies are carried out in this area. For example, different sizes of TiO_2_-NPs were synthesized by using betel pepper leaves, aloe vera paste [32], and hay bacillus (Bacillus subtilis] bacteria [33] as shown in various studies [34]. Another parameter of the green synthesis method of titanium dioxide nanoparticles is the selection of the initiator. Four different initiators are used for this. These are titanium tetraisopropoxide (TTIP) [35], titanium chloride (TiCl_4_) [36], tetra-n-butyl orthotitanate (TBT) [13], and commercial titanium dioxide powder [37]. Nanoparticles between 1–100 nm are synthesized with all these initiators. TTIP, which is used in the classical sol-gel method, is the most used initiator in the green synthesis method [38]. The starting solvents and particles are given in Tables (1–3), respectively.[Table t1-turkjchem-46-5-1345][Table t2-turkjchem-46-5-1345][Table t3-turkjchem-46-5-1345]

Rueda et al. [35] prepared the extract of 50 g of tangerine peel selected as a solvent with 150 mL of distilled water for 2 h on a 400-rpm magnetic stirrer at 90 ± 3 °C. They would prepare a 1.5 Normality TTIP solution and mix it with tangerine peel extract in 2 different proportions. A type of sample was prepared by mixing 68 mL of TTIP with 1 mL of extract, and a B type sample by mixing 68 mL of TTIP and 55 mL of extract was prepared in two solutions at pH 5. These two sample types were mixed and centrifuged at 700 rpm for 3 h. The precipitated samples were dried at 100 °C for 8 h and calcined at 600 °C for 3 h. It was observed that nanocrystals between 50 and 150 nm were formed in these two samples, and there was only a significant difference between the cluster sizes. A-type nanocrystals clustered at 700 nm, while B-type nanocrystals clustered at 350 nm.

Irshad et al. [38] synthesized TiO_2_ nanoparticles with Trianthema portulacastrum and Chenopodium quinoa plants. They washed the leaves thoroughly with deionized water and left them to dry for a few days. They were powdered with a blender in dry water and kept in deionized water for 36 h. They used titanium tetra isopropoxide as an initiator. By mixing 1:2 ratios with solvent/initiator balance, a hydrolysis reaction was achieved, and the product was calcined at 450 °C. Plants were used as solvents and replaced Tween-80, isopropyl alcohol, and acidic acid in the sol-gel method. They synthesized another TiO_2_-NP with these chemicals. The sizes of TiO_2_ nanoparticles synthesized by the chemical sol-gel method are around 10–13 nm, while the sizes of those synthesized by the green synthesis method are around 6–8 nm. Wherein the particle sizes are calculated using the Scherrer equation. Kashale et al. [26] synthesized a titanium dioxide nanoparticle with chickpea (Cicer arietinum L.). Twenty g of dry chickpea leaves were kept in 100 mL of deionized water for 6 h in the room and were filtered. It was completed to 50 mL by mixing 10 mL of TiCl_4_ with 10 mL of chickpea extract. Ammonia was added until the pH reached 7, then it was calcined at 500 °C, the organic compound was liberated, and nanoparticles were obtained. They could synthesize titanium dioxide nanoparticles of approximately 14 nm in size.

Madadi et al. [13] synthesized titanium dioxide nanoparticles using licorice. After washing and drying the licorice root, it was cut into small pieces and degreased with acetone and methanol in the soxhlet device. It was dried in air and extracted with methanol in a soxhlet apparatus. Then, together with centrifugation, the extract was dried in a flash evaporator. Tetra-n-butyl orthotitanate (TBT) was used as an initiator and passed through classical sol-gel methods to form titanium dioxide nanoparticles. A material diameter of 24 nm was obtained from the XRD results.

Saranya et al. [41] synthesized TiO_2_-NP with Cochlospermum Gossypium with a titanium oxysulphate initiator. They added 50 mg of Cochlospermum Gossypium to 10 mL of 0.1 M titanium oxysulphate and mixed it at 750 rpm at 90–95 °C. The extraction was centrifuged, washed, and dried. Then, it was calcined at 500 °C for 4 h, and the product was obtained. This synthesis method received titanium dioxide nanoparticles between 8 and 13 nm with the anatase crystal structure.

## 4. Applications of titanium dioxide nanoparticles

### 4.1. Dye removal applications with titanium dioxide nanoparticles synthesized with renewable resources

**Figure 3:**
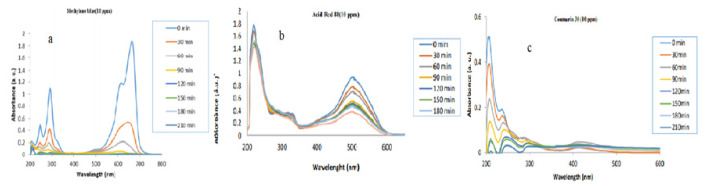
Titanium dioxide nanoparticles synthesized with licorice a) Removal of methylene blue b) Acidic red removal of 88 c) Removal of coumarin 80 [48].

Madadi et al. [13] applied the removal of methylene blue, acidic red 88, and coumarin 30 dyes with titanium dioxide nanoparticles synthesized with licorice root. TiO_2_-NP (0.03 g) was mixed with dyes in 10 mg/L and 20 mg/L in 500 mL. It was ultrasonically mixed for 5 min and left in the dark for 30 min. The stock solutions were then incubated under a UV lamp (250 W high), and 3 mL were taken every 30 min. Samples were centrifuged at 13,000 rpm. Liquid solutions were analysed in the UV spectrophotometer. Figure 3 shows us a 30-min reduction of dyes in the UV spectrophotometer. As a result, titanium dioxide nanoparticles synthesized with green synthesis demonstrated successful results in methylene blue.

**Figure 4:**
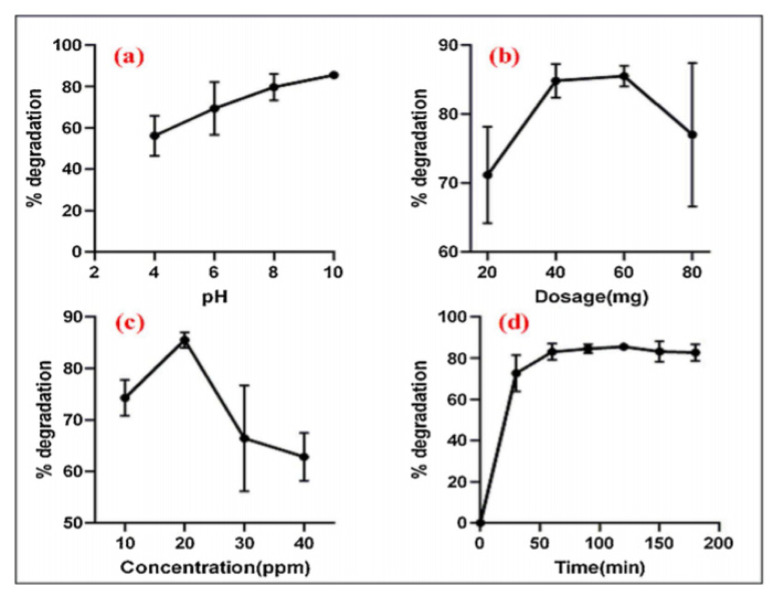
Removal of methylene blue dye with titanium dioxide synthesized with Monsonia burkeana plant a) pH b) dosage c) concentration d) time [47].

Ngoepe et al. [47] synthesized titanium dioxide nanoparticles with the Monsonia burkeana plant and used these nanoparticles for methylene blue dye removal. The sizes of the nanoparticles are between 6–10 nm. For the UV lamp, 300 W Ultra-Vitalux was preferred. pH and dosage parameters were applied in dye removal. Using Equation 9, they calculated the % degradation values in the ordinates given in Figure 4.


(9) $$C=(\frac{C_{0}-C}{C_{0}})*100$$

**Figure 5:**
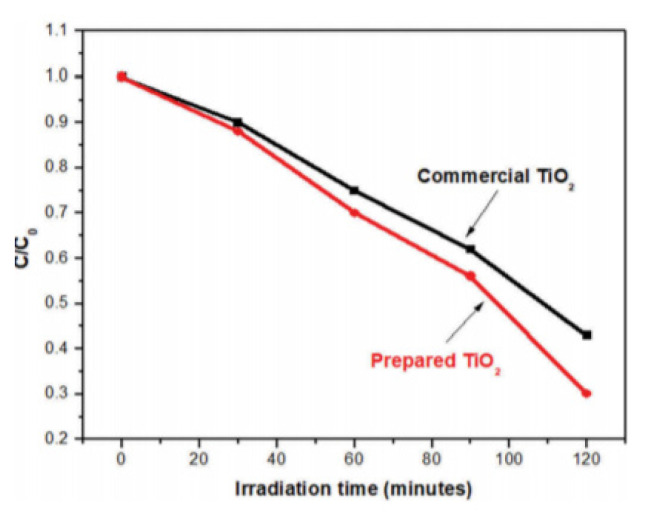
Removal of Rhodamine B dye with titanium dioxide synthesized with lemon peel extract [45].

Nabi et al. [45] studied the removal of Rhodamine B dye with titanium dioxide. It synthesized sizes between 80–120 nm, which synthesized titanium dioxide nanoparticles with lemon peel extract. These nanoparticles produced better results than commercially available titanium dioxide. As can be seen in Figure 5, it is provided for a dye removal of nearly 70% as in the C/C0 graph against irradiation time.

**Figure 6:**
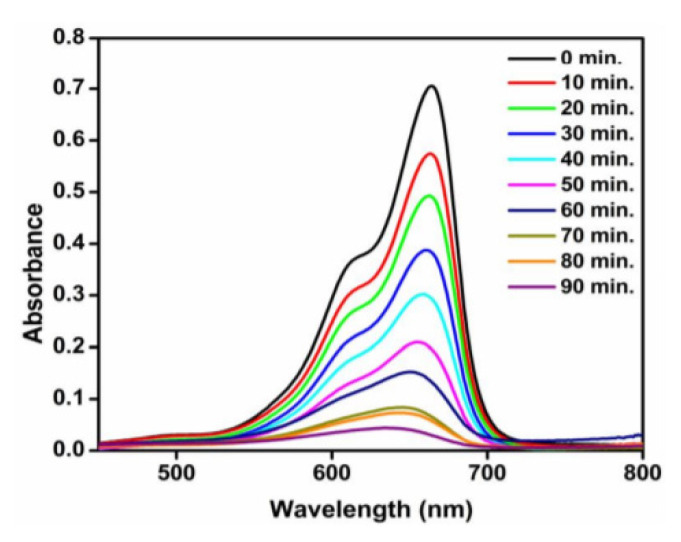
Removal of methylene blue dye with titanium dioxide synthesized titanium oxysulfate [41].

TiO_2_-NPs (anatase, 8–13 nm) synthesized with Titanium oxysulfate and Cochlospermum Gossypium removed organic dyes. Saranya et al. [41] added 10 mg of TiO_2_-NP to 50 mL of dye solution (1.0 × 10^−5^ M) and kept it in a stirred media at 600 rpm under the sunlight. The absorption value was measured in UV-spectrophotometer by taking 5 mL of the sample every 10 min. As a result, dye removal was seen in Figure 6, with samples taken in 10-min periods from the methylene blue dye mixture.

### 4.2. Dye removal applications with titanium dioxide nanoparticles synthesized with chemical methods

**Figure 7:**
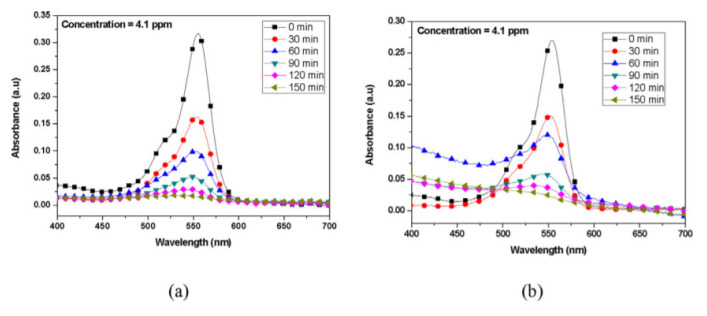
Absorption Spectrum of Rhodamine B dye degraded by (a) sol-gel prepared catalyst and (b) Hydrothermally prepared catalyst [48].

**Figure 8:**
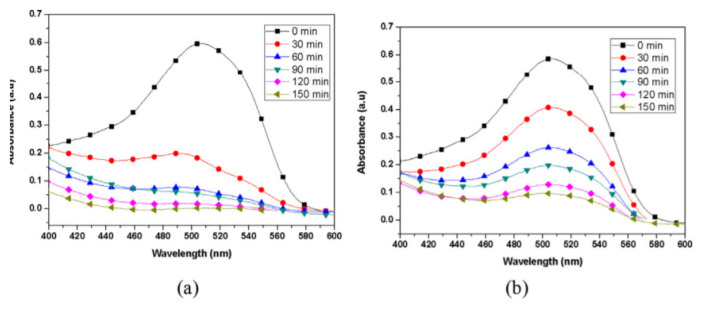
Absorption Spectrum of Sudan III dye degraded by (a) Hydrothermal prepared TiO_2_ catalyst, and (b) Sol-gel prepared TiO_2_ catalyst [48].

Dodoo-Arhin et al. [48] made comparative dye removal applications with two different titanium dioxide nanoparticles synthesized by the sol-gel and hydrothermal methods. They chose TCl4 for the hydrothermal method and TTIP initiator for the sol-gel process. They were carried out under a 330W UV lamp. Rhodamine B dye (0.01 g) was mixed with 200 mL of distilled water to obtain the dye solution. To obtain the TiO_2_ suspension, 0.1 g of the as-prepared TiO_2_ nanoparticles were mixed with 50 mL of distilled water in a different beaker. Thirty mL of the dye solution was then added to the prepared TiO_2_ suspension. In the Sudan III dye degradation experiment, a stock solution was prepared by dissolving 0.8 g of Sudan III dye in 400 mL of Isopropyl alcohol under sonication for about 30 min. The solution was filtered, and the filtrate was used as the dye solution for the photodegradation experiment. Fifty mL of the as-prepared Sudan III dye solution was mixed with 0.1 g of TiO_2_ catalyst to obtain Sudan III dye. In the graphics in Figure 7 and Figure 8, the removal of these dyes based on time is given. As a result, it has been observed that titanium dioxide nanoparticles synthesized by the sol-gel method are more efficient in dye removal.

### 4.3. Wastewater cleaning applications with titanium dioxide nanoparticles synthesized with renewable resources

**Figure 9:**
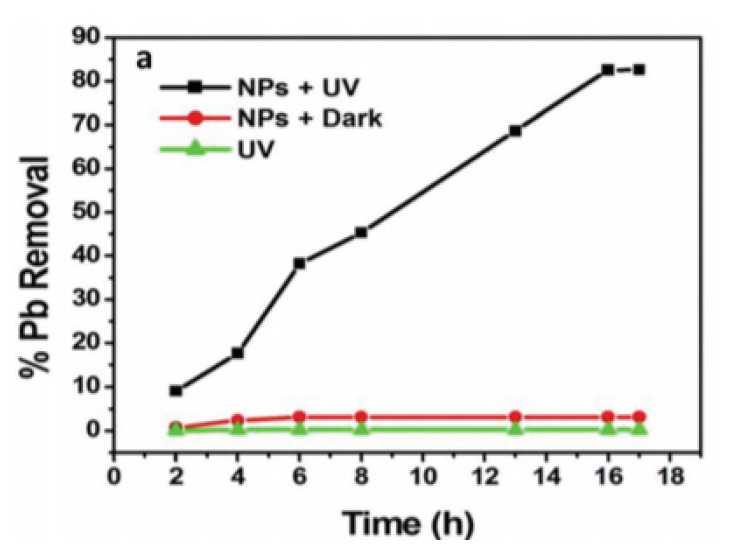
Removal of lead in the wastewater with titanium dioxide nanoparticles synthesized from java plum leaves [27].

Sethy et al. [27], in their study in 2020, investigated the photocatalytic performance of titanium dioxide nanoparticles synthesized from java plum (black plum) leaves on explosive industrial wastewater containing lead pollutants. Wastewater samples were collected and stored between 6 and 7 °C. For 500 mL of wastewater, 0.3 g of green titanium dioxide nanoparticles were used and kept under a 15 W UV lamp for 12 h. At the same time, another solution was prepared, which was not kept under the UV lamp. It was then centrifuged at 9000 rpm for 10 min, and precipitates were collected. Inductively Coupled Plasma Atomic Emission Spectroscopy measured lead concentration in wastewater. The percentage reduction is given in Figure 9. As a result, green TiO_2_-NPs become photocatalytically active, and they can reduce lead in wastewater.

Goutam et al. [49] studied tannery wastewater cleaning with titanium dioxide nanoparticles synthesized with the Jatropha curcas L. plant. They determined that they synthesized 13 nm size titanium dioxide nanoparticles calculated from the XRD results. The wastewater collected from the water treatment outlet of the tannery was stored. Then the system was prepared with 5 g of titanium dioxide nanoparticles for 5 L wastewater and left under sunlight for 5 h. After waiting for 15 min, the titanium dioxide was allowed to settle, and samples were taken for chemical oxygen demand (COD) and Cr tests. As a result, it was seen that 82.26% COD and 76.48% Cr were removed. This study on tannery wastewater concluded that the titanium dioxide nanoparticles synthesized from the plant Jatropha curcas L. are environmentally friendly.

### 4.4. Wastewater cleaning applications with titanium dioxide nanoparticles synthesized by chemical method

**Figure 10:**
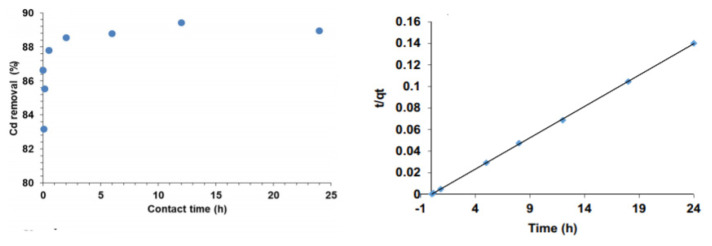
% Removal of cadmium in wastewater by titanium dioxide nanoparticles [38].

Irshad et al. [38] studied titanium dioxide nanoparticles synthesized by the classical sol-gel method to remove cadmium from wastewater. A stock solution of 3000 mg/L was prepared with 1 L of Cd (NO_3_)_2_, 4H_2_O salt, and 1 L of deionized water. Wastewater removal by batch sorption method was investigated. Twenty-five mL of cadmium water in 50 mL plastic tubes was taken from the stock solution. Different pH parameters were established with 0.1 M NaOH/HCl solutions. The effect of pH (3–10) on Cd adsorption was examined by employing 25 mL of batch sorption solution at 0.7 g/L sorbent dosage with 30 mg/L, for 2h shaking period. As a result, pH 4.3, sorbent dose 0.7 g/L, initial concentration of Cd 30 mg/L, and contact time 2 h for sodium absorption were obtained for optimum conditions. % Cd removal graph is given in Figure 10.

### 4.5. Antibacterial studies with titanium dioxide nanoparticles synthesized with chemical methods

**Figure 11:**
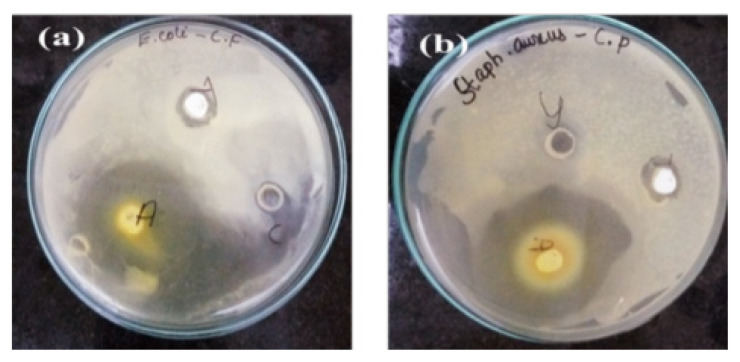
Effect of titanium dioxide nanoparticles synthesized with Cassia fistula plant on a) E. Coli b) S. aureus bacteria [46].

Swathi et al. [46] synthesized titanium dioxide nanoparticles using Cassia fistula leaves. They carried out the removal of gram-positive and gram-negative bacteria with nanoparticles. It was tested on Escherichia coli and Staphylococcus aureus bacteria by performing the antibacterial study with the “agar well diffusion” method. As seen in Figure 11, they achieved successful results on both bacteria.

Kansal and his students [50] compared the antibacterial properties of titanium nanomaterials synthesized with green synthesis and chemically synthesized titanium dioxide nanomaterials. Hibiscus Rosa Sinensis was used as an extraction. As a result, it was observed that it was smaller and more scattered than nanomaterials synthesized by chemical methods. Titanium dioxide nanoparticles synthesized with flower extract exhibited considerable antimicrobial activity against pathogenic bacteria. It was predicted that green synthesized titanium dioxide nanoparticles can be used in potential biomedical applications compared to chemically synthesized TiO_2_ nanoparticles due to their dispersibility, stability, and surface coatings.

### 4.6. Lithium battery applications with titanium dioxide nanoparticles synthesized with chemical methods

Kashale et al. [26], in their study in 2016, used titanium dioxide nanoparticles synthesized with chickpea plants in lithium-ion battery applications. For electrochemical measurements, lithium metal was used for both the reference and counter electrode. The composite electrode was prepared with 10 mg of active material (TiO_2_ NP), 1.5 mg of conductive material (super P), and 1.5 mg of tetanized acetylene black (TAB-2) as the binder. The application was tested using a tester with a constant current density of 33 mA/g. As a result, titanium dioxide nanoparticles synthesized with a green synthesis that does not contain aggregation increased the surface area, increased the electrolyte/electrode contact area, shortened the path lengths for both Li-ion and electron transport, and reduced specific current density. The synthesis of these electrodes significantly improved cell performance and cycle stability.

### 4.7. Solar cell applications with titanium dioxide nanoparticles synthesized with chemical methods

In the article published by Maurya et al. [3] in Solar Energy magazine, they compared titanium dioxide powders used in the industry in solar cells with 9–13 nm titanium dioxide nanoparticles sizes synthesized with fresh Bixa Orellana seeds. A high increase in the power conversion efficiency of the solar cell was achieved when green synthesized titanium dioxide was used as a photoanode. It was observed that these green synthesized mesoporous titanium dioxide nanofilms had higher photocurrent density than industrial using titanium dioxide powders.

### 4.8. Anticancer studies with titanium dioxide nanoparticles synthesized with renewal methods

**Figure 12:**
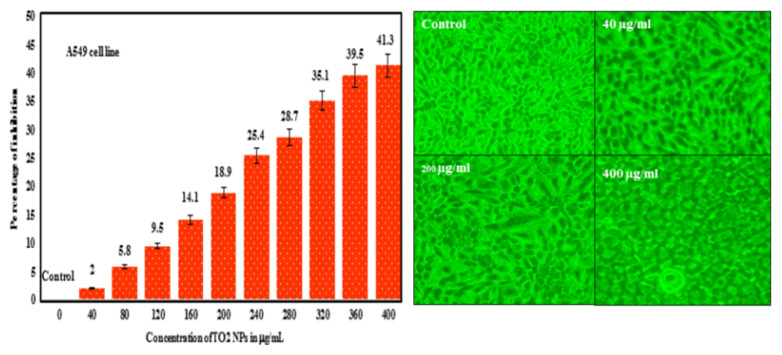
TiO_2_-NPs’ per inhibition/concentration in A549 cell [42].

Amanulla and Sundaram [42] studied the inhibition of cancer cells by titanium dioxide nanoparticles synthesized with the extract of orange peels. The titanium dioxide nanoparticles they synthesized in 17.30 and 21.61 nm sizes were formed in anatase. The in vitro cytotoxicity of these nanoparticles was done in the A549 cell line using the “3-(4,5-Dimethylthiazol-2-yl)-2,5-Diphenyltetrazolium Bromide” (MTT test). TiO_2_-NPs synthesized by green synthesis exhibited 41% inhibition at a concentration of 400 μg/mL. Biomolecules found in orange peel extract produce electrons, increasing reactive oxygen species (ROS) production on the surface of cancer cell lines preferred for cell death. Cell viability gradually decreased with increasing TiO_2_-NP concentration. The inhibition/concentration graph is given in Figure 12.

### 4.9. Antifungal application with titanium dioxide nanoparticles synthesized with renewable methods

**Figure 13:**
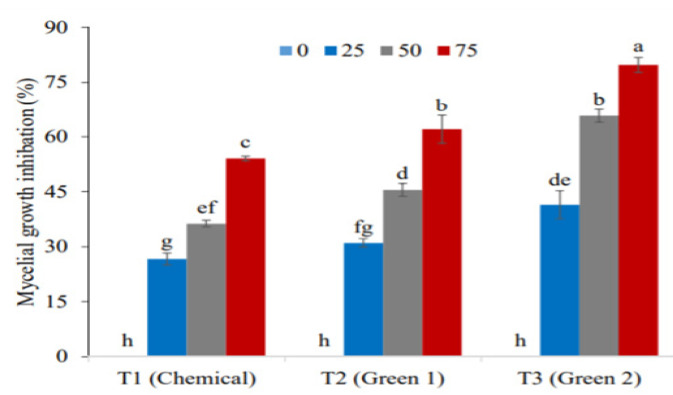
Antifungal effect of titanium dioxide nanoparticles synthesized by green synthesis against Wheat Rust fungus. T1: chemical method, T2 Green 1: method with T. portulacastrum plant, T3 Green 2: method with C. quinoa plant [51].

Titanium dioxide nanoparticles synthesized with Trianthema portulacastrum and Chenopodium quinoa plants showed antifungal properties. Irshad and his students [51] tested the preventive effects on Wheat Rust mushrooms in 2020. Concentrations (0.10 mg/mL NP into 25μL, 50μL, and 75μL) were prepared and mixed in Potato Dextrose Agar (PDA), which growing medium, and then 20 mL of this medium were poured into sterile Petri plates with a diameter of 90 mm. Petri plates were left overnight, and a culture medium was created. Petri plates were covered with paraffin and incubated at 25 °C ± 2 for 5 days. Figure 13 shows the graph of mycelial growth inhibition. In this case, they observed that titanium dioxide nanoparticles synthesized with the Chenopodium quinoa plant are good antifungal agents.

## 4. Conclusion

It is seen in this review that TiO_2_ nanoparticles synthesized using the green synthesis method, which is an easy and inexpensive method, are more functional than other TiO_2_ nanoparticles synthesized by the chemical method. It is seen that TiO_2_ nanoparticles synthesized with plant extracts have smaller sizes and relatively higher photocatalytic properties compared to TiO_2_ nanoparticles synthesized by the chemical sol-gel method. In this way, instead of chemicals as waste in nature, there will be natural wastes that can be destroyed more quickly.

Waste chemical dyes and solvents, which are starting to destroy natural resources day by day, are seen as a waste of electricity and time to be physically treated. Titanium dioxide nanoparticles can be preferred due to the advantage of being used as a photocatalyst and being reusable without harming nature. Other metal oxide nanoparticles of different UV-wavelengths can also be used instead of titanium dioxide nanoparticles. For example, zinc oxide, iron oxide, magnesium oxide, and tungsten oxide nanoparticles can exhibit photocatalytic properties at their wavelengths, just like titanium dioxide nanoparticles.

There are many plant species with high -OH bonds in nature. Plants with hydroxyl bonds such as orange peels, olive leaves, and licorice roots, which are seen as waste, can be collected and used in the synthesis of many different nanoparticles. Thus, it is possible to find a way to get rid of chemicals that will not be lost in nature or that will harm living things.

Different industries that generate waste dye or organic waste, such as the textile industry, are increasing in Turkey. When we consider the wastes left to nature, it becomes clear that we need a new idea. For this purpose, it is important to produce effective, reusable, cheap but powerful photocatalysts using these wastes.

## Figures and Tables

**Table 1 t1-turkjchem-46-5-1345:** TiO_2_-NPs synthesized by green synthesis with TTIP initiator.

Renewable resource	Type	Crystal size (nm)	Writer
Nyctanthes Arbor-tristis L	Leaves	~100	Sundrarajan et al. 2011 [39]
Acanthophyllum Laxiusculum	Root	25	Madadi et al. 2016 [13]
Chenopodium quinoa	Leaves	0–50	Irshad et al. 2020 [38]
Phyllanthus niruri	Leaves	32	Shanavas et al. 2019[37]
Citrus reticulata	Peel	50–100	Rueda et al. 2020 [35]
Syzygium cumini	Leaves	10	Sethy et al. 2020 [27]
Cymbopogon citratus	Leaves	10	Solano et al. 2019 [40]

**Table 2 t2-turkjchem-46-5-1345:** TiO_2_-NPs synthesized by green synthesis with TiCl_4_ initiator.

Renewable Resource	Type	Crystal size (nm)	Writer
Cicer arietinum L.	Leaves	14	Kashale et al. 2016 [26]
B. Variegata	Leaves	3–8	Maurya et al. 2012 [3]
Aloe Vera	Leaves	20–90	Rao et al. 2015 [32]
Citrus sinensis	Peel	21–61	Amanulla et al. 2018 [42]

**Table 3 t3-turkjchem-46-5-1345:** TiO_2_-NPs synthesized by green synthesis with TiO_2_ Powder initiator.

Renewable Resource	Type	Crystal size (nm)	Writer
Echinacea purpurea	Leaves	120	Dobrucka, 2015 [43]
Moringa oleifera	Leaves	100	Sivaranjania et al. 2015 [44]
Azadirachta indica	Leaves	25–85	Thakur et al. 2019 [36]
Citrus limon	Peel	80–120	Nabi et al. 2020 [45]
Cassia fistula	Leaves		Swathi et al. 2019 [46]
